# Indigenous rights, health and traditional medicine systems

**DOI:** 10.2471/BLT.24.292882

**Published:** 2025-09-22

**Authors:** Nicole Redvers, Kelly Menzel, Danya Carroll, Carol Zavaleta, Sylvia Kokunda, Daniel M Kobei, Juan Nelson Rojas

**Affiliations:** a Schulich School of Medicine and Dentistry, University of Western Ontario, 1465 Richmond Street, London, ON, N6G 2M1, Canada.; b Burnet Institute, Melbourne, Australia.; c Intercultural Citizenship and Indigenous Health Unit (UCISI), Cayetano Heredia University, Lima, Peru.; d Action for Batwa Empowerment Group, Buhoma, Uganda.; e Ogiek Peoples’ Development Program, Egerton, Kenya.; f Pipil Indigenous Council of Firekeepers and Healers, Santa Tecla, El Salvador.

## Abstract

The health of Indigenous Peoples around the world is tied to longstanding Indigenous knowledge practices and access to Indigenous traditional medicine systems. Indigenous communities continue to have concerns, however, about their ability to ensure intergenerational transfer of Indigenous traditional medicine knowledge. These concerns stem from the continued lack of Indigenous rights as a result of colonization, a lack of protection of land rights and different levels of Indigenous self-determination around the world, all of which affect traditional medicine access and practices. Indigenous Peoples’ access to their traditional medicine systems will therefore continue to depend on their access to self-determination, land and cultural rights, as well as on Indigenous data sovereignty. A balanced approach to Indigenous health and well-being is needed that prioritizes Indigenous traditional practices, community involvement, and Indigenous cultural and language revitalization (with support for ongoing intergenerational knowledge transfer). These elements are important for bringing back ecological harmony to Indigenous Peoples, communities and the planet. Additionally, with sufficient resources and support, Indigenous Peoples and their concepts of health can be a blueprint for harmony for people and health systems more widely, with Indigenous Peoples being a key determinant of planetary health. We outline some key points to ensure that the concepts of health and well-being of Indigenous Peoples and their Indigenous traditional medicine systems are maintained going forward for individual, community and planetary health.

## Résumé

La santé des peuples autochtones à travers le monde est liée aux pratiques ancestrales issues des savoirs autochtones et à l’accès aux systèmes de médecine traditionnelle autochtone. Les communautés autochtones continuent toutefois de s’inquiéter de leur capacité à assurer la transmission intergénérationnelle des connaissances en matière de médecine traditionnelle autochtone. Ces préoccupations découlent de l’absence persistante de droits autochtones résultant de la colonisation, du manque de protection des droits fonciers et des différents niveaux d’autodétermination des peuples autochtones à travers le monde, qui ont tous une incidence sur l’accès à la médecine traditionnelle et ses pratiques. L’accès des peuples autochtones à leurs systèmes de médecine traditionnelle continuera donc de dépendre de leur accès à l’autodétermination, aux droits fonciers et culturels, ainsi qu’à la souveraineté des données autochtones. Une approche équilibrée de la santé et du bien-être des peuples autochtones est nécessaire, qui donne la priorité aux pratiques traditionnelles autochtones, à la participation communautaire et à la revitalisation de la culture et de la langue autochtones (avec un soutien au transfert intergénérationnel continu des connaissances). Ces éléments sont importants pour rétablir l’harmonie écologique des peuples autochtones, des populations locales et de la planète. De plus, moyennant des ressources et un soutien suffisants, les peuples autochtones et leurs concepts de santé permettent de servir de modèle d’harmonie pour les populations et les systèmes de santé à plus grande échelle, les peuples autochtones étant un facteur déterminant de la santé planétaire. Nous soulignons quelques points clés afin de garantir la préservation des concepts de santé et de bien-être des peuples autochtones et de leurs systèmes de médecine traditionnelle en faveur de la santé des individus, des populations locales et de la planète.

## Resumen

La salud de los pueblos indígenas de todo el mundo está estrechamente vinculada a las prácticas ancestrales de conocimiento indígena y al acceso a los sistemas de medicina tradicional indígena. No obstante, las comunidades indígenas continúan expresando su preocupación por su capacidad para garantizar la transmisión intergeneracional de los conocimientos sobre medicina tradicional indígena. Estas inquietudes se derivan de la persistente falta de reconocimiento de los derechos de los pueblos indígenas como consecuencia de la colonización, de la ausencia de protección de los derechos sobre la tierra y de los distintos niveles de autodeterminación indígena existentes en el mundo, factores que afectan al acceso y las prácticas relacionadas con la medicina tradicional. Por consiguiente, el acceso de los pueblos indígenas a sus sistemas de medicina tradicional seguirá dependiendo de su derecho a la autodeterminación, del acceso a los derechos territoriales y culturales, así como de la soberanía de los datos indígenas. Es necesario un enfoque equilibrado de la salud y el bienestar indígenas que priorice las prácticas tradicionales, la participación comunitaria y la revitalización cultural y lingüística indígena (con apoyo a la transmisión intergeneracional continua del conocimiento). Estos elementos resultan esenciales para restablecer la armonía ecológica de los pueblos indígenas, sus comunidades y el planeta. Además, con recursos y apoyo adecuados, los pueblos indígenas y sus concepciones de la salud pueden servir de modelo de armonía para las personas y los sistemas sanitarios en general, siendo los pueblos indígenas un factor determinante clave de la salud planetaria. En este artículo, se exponen los puntos fundamentales para garantizar que las concepciones de salud y bienestar de los pueblos indígenas, así como sus sistemas de medicina tradicional, se mantengan en el futuro para el beneficio de la salud individual, comunitaria y planetaria.

## ملخص

ترتبط صحة الشعوب الأصلية حول العالم بالممارسات المعرفية الأصلية الراسخة، وإمكانية الوصول إلى أنظمة الطب التقليدي الأصلية. ومع ذلك، لا تزال مجتمعات الشعوب الأصلية تشعر بالقلق بخصوص قدرتها على ضمان نقل معارفها في الطب التقليدي الأصلي عبر الأجيال. تنتج هذه المخاوف من الافتقار المستمر لحقوق الشعوب الأصلية نتيجة للاستعمار، وعدم حماية حقوقها في الأراضي، والمستويات المختلفة في حقوق تقرير المصير حول العالم، وكلها عوامل تؤثر على إمكانية الوصول إلى الطب التقليدي وممارساته. وبالتالي سيظل وصول الشعوب الأصلية إلى أنظمة الطب التقليدي الخاصة بها، معتمدًا على إمكانية وصولها إلى حق تقرير المصير، وحقوقها في الأراضي وحقوقها الثقافة، بالإضافة إلى سيطرة البيانات الأصلية. هناك حاجة إلى أسلوب متوازن لصحة الشعوب الأصلية ورفاهيتها، بحيث يتم منح الأولوية للممارسات التقليدية للشعوب الأصلية، والمشاركة المجتمعية، وإعادة إحياء ثقافتها ولغتها (مع دعم النقل المستمر للمعرفة عبر الأجيال). هذه العناصر مهمة لإعادة التناسق البيئي للشعوب الأصلية، ومجتمعاتها، وكوكب الأرض. بالإضافةً إلى ذلك، مع توفر الموارد والدعم الكافيين، فإنه يمكن للشعوب الأصلية ومفاهيمها الصحية أن تُشكل نموذجًا يُحتذى به لتحقيق التناسق بين الناس والأنظمة الصحية على نطاق أوسع، مع اعتبار الشعوب الأصلية عاملًا أساسيًا في صحة الكوكب. نحن نحدد بعض النقاط الرئيسية لضمان الحفاظ على مفاهيم الصحة والرفاهية للشعوب الأصلية وأنظمة الطب التقليدي الخاصة بها مستقبلًا، بما يضمن صحة الفرد والمجتمع والكوكب.

## 摘要

世界各地土著人民的健康与由来已久的土著知识实践和土著传统医药体系的获取途径密切相关。不过，土著人民社区也仍然担心他们是否有能力确保将他们的土著传统医药知识传给下一代。这些担忧源自其因为殖民统治而导致的持续缺乏土著人民权利、无法保护其土地权利，以及世界各地土著人民自决权发展水平参差不齐，所有这些因素都影响着传统医药的获取和践行。因此，土著人民对其传统医药体系的获取将仍取决于他们是否有自决权、土地和文化权，以及土著人民是否有数据主权。土著人民的健康和福祉需要一种平衡之法，该方法优先关注土著人民传统实践、社区参与和土著人民文化和语言复兴（并为持续的代际知识传承提供支持）。这些要素对重现土著人民、社区和整个星球的生态和谐至关重要。另外，有了充足的资源和支持，土著人民及其健康理念能从更广泛的程度为人们和健康体系的和谐绘制蓝图，土著人民是我们星球健康的关键决定因素。我们概述了一些关键要点，以确保为了个人、社区和我们星球的健康，土著人民的健康和福祉理念及其传统医药体系在未来能得以维持。

## Резюме

Здоровье коренных народов во всем мире связано с их давними практиками, знаниями и доступом к системам традиционной медицины. Однако коренные сообщества озабочены тем, смогут ли они и далее передавать из поколения в поколение знания, относящиеся к традиционной медицине коренных народов. Эти опасения проистекают из продолжающегося ущемления прав коренных народов в результате колонизации, в недостаточной защите прав на землю и в различии уровней самоопределения тех или иных народов как коренных в различных странах мира. Все эти факторы влияют на доступ к традиционной медицине и на соответствующие практики. Таким образом, доступ коренных народов к их системам традиционной медицины будет и впредь зависеть от их доступа к правам на самоопределение, землю и культуру, а также от суверенности сведений, принадлежащих коренным народам. Сбалансированный подход к здоровью и благополучию коренного населения – это тот подход, в котором первоочередную важность приобретают традиционные практики коренного населения, вовлеченность общественности, возрождение языков и культуры коренных народов (с поддержкой непрерывной передачи знаний из поколения в поколение). Эти элементы важны для возрождения экологической гармонии коренных народов, общин и планеты в целом. Кроме того, при наличии достаточных ресурсов и поддержки коренные народы и их концепция здравоохранения могут стать основой для более широкой гармонии для людей и систем здравоохранения, в которых коренное население станет ключевой детерминантой здоровья во всем мире. Авторы очерчивают некоторые ключевые позиции, которые позволят сохранить существование и развитие концепций здоровья и благополучия коренных народов и их систем традиционной медицины с целью сохранения здоровья каждого человека, сообществ и планеты в целом.

## Introduction

The health of Indigenous Peoples in many communities around the world has been tied to intergenerational Indigenous knowledge transfer and access to Indigenous traditional medicine systems that continue to contribute to the well-being of individuals and families. Indigenous traditional medicine systems have a deep interconnectivity to the lands and waters where Indigenous Peoples live. Despite the direct importance of these traditional medicine systems to the health and well-being of Indigenous Peoples, these systems have been subject to discrimination,^1^ misappropriation^2^ and deterioration due to environmental destruction.^3^^,^^4^ This situation is a direct result of colonialism and the ensuing epistemicide^5^ (the killing, silencing, annihilation or devaluing of knowledge systems),^6^ with current biomedical models being consequently based on policy frameworks (for example, government and health insurance regulations, clinical guidelines and corporation incentives from the global pharmaceutical industry) that lead to and benefit from the annihilation of Indigenous ancestral knowledges and wisdom.

Despite generations of human rights abuses perpetrated on Indigenous Peoples, including the ongoing forced removal from traditional homelands;^7^ discriminatory child welfare policies;^8^ the loss of Indigenous languages; and continuing colonial assimilation policies that denigrate Indigenous ways of knowing and being,^9^ many Indigenous communities continue to steward their traditional medicine systems. Nevertheless, Indigenous communities are concerned about their ability to ensure intergenerational transfer of Indigenous traditional medicine knowledges, given the lack of protection for land rights and the differing levels of Indigenous self-determination around the world. Indigenous Peoples continue to demonstrate great resilience in upholding their holistic approaches to health, which integrate an understanding of the need for a dynamic balance of well-being that includes the spiritual, emotional, mental and physical realms, as well as the health of Mother Earth. This view has often been at odds with more conventional biomedical notions of health and well-being that are devoid of any consideration for planetary health and well-being.^10^ In this paper, we contend that Indigenous Peoples and their traditional medicine systems are fundamentally connected to important past and current geopolitical factors that cannot be minimized.

### Position of the authors

We are a group of Indigenous authors representing different nations from around the world: Deninu K’ue First Nation (Canada); Bundjalung Nation (Australia); Diné and White Mountain Apache Tribes (United States of America); Quechua Peoples (Peru); Batwa Peoples (Uganda); Ogiek Peoples (Kenya); and Nahua Peoples (El Salvador). Our perspectives come from our own lived experiences connected with and extended through different national and international work that amplifies Indigenous voices on this topic. We position ourselves within our Indigenous communities, as per Indigenous protocol, to situate our voices and the positions we take in this article.^11^ We also outline throughout this paper important concepts identified through our work that we see as relevant, and attempt to amplify Indigenous voices from other contexts. We are mindful, however, that our views come from our own experiences and contexts. Lastly, we do not promote single definitions of concepts (for example, Indigenous traditional medicine) due to the vast diversity of Indigenous Peoples around the world which is important to respect.^12^ Thus, variation in the use of terms is purposeful and depends on the geopolitical context in which the terms are introduced.

## Partnership or integration?

Indigenous Peoples and their knowledges have typically been excluded from health and well-being frameworks that guide national or international policy-setting in health. Additionally, medical and health-related curriculums for health professionals most often exclude cultural safety (that is, a critique of power imbalances and critical self-reflection)^13^ or implement it very poorly. Yet, if cultural safety were included sufficiently, it could support a better understanding of the health needs of Indigenous Peoples, including their medicine systems, and support more informed policy. There are, from our perspective, justified sensitivities about the sharing of Indigenous traditional medicine knowledges outside of Indigenous communities themselves. However, the ability of health systems and health professionals to be culturally responsive to the needs and self-determination of Indigenous Peoples is critical to improving their health outcomes.^14^ Additionally, there has been increasing interest in Indigenous knowledges in certain health-related areas including biodiversity conservation, climate change and health, as well as the use of some plant-based medicines (e.g. psychedelic/Sacred medicines).^15^^,^^16^ However, this interest has most often been from the perspective of how Indigenous Peoples and their knowledges could be integrated into Euro-Western biomedical models of health and the delivery of health care (that is, European or Western-centric worldviews and practice regardless of current geography). This focus is problematic for several reasons as highlighted by the following quotation from a 2019 article:

The noted restraints and accumulated barriers experienced in developing Indigenous wellness services can be rooted in the effort of trying to fit an Indigenous model into a non-Indigenous…[Euro-Western-centric]…system. Indigenous culturally based interventions are often defined by a Western methodological approach, governance structure, and fiscal reporting requirement which constrains and changes the programme itself. Utilizing an Indigenous methodological approach, governance structure and reporting approach, and thereby adapting the…[Euro-Western-centric]…system to this approach and structure, would be a truly self-determined model with a higher degree of success.^17^

Creating bridges between, partnering with or supporting Indigenous Peoples and their ability to use and have access to their traditional medicine systems may be more appropriate than assuming that integration is desired or acceptable, given past and current experiences of discrimination. Integration can be seen as a modern form of colonial assimilation if done without appropriate safeguards in place. Indigenous traditional medicine systems also risk being culturally appropriated without consideration of their relevance. This situation could lead to Indigenous medicine systems being reduced to a superficial tourist practice, where most of the benefits are directed to external audiences at the cost of Indigenous Peoples themselves, and without individuals from the outside having to deeply engage with the epistemological underpinning (that is, the theory of knowledge) of Indigenous knowledges.^18^^,^^19^ Additionally, as Indigenous traditional medicine systems are deeply connected to spiritual ways of being in the world, economic or business connectivity (for example, economic benefit) can be a problematic approach for many Indigenous communities. Past and current experiences exist of pharmaceutical drugs being derived from the medicinal knowledge of Indigenous Peoples without their consent, or the commercial benefits not being given back to communities. As a result, Indigenous communities justifiably lack trust in corporate and research entities.^16^^,^^20^ Many Indigenous communities are grounded in oral knowledge systems and have strict cultural protocols in place for how medicine knowledge is handled and passed on. Therefore, moving towards more modern models with formal written records, which are standard in research or corporate involvement, also produces challenges related to Indigenous data sovereignty and the protection of Indigenous knowledges.

## Stewardship of Indigenous data 

Indigenous data sovereignty and Indigenous data governance are essential elements to retain the integrity of Indigenous knowledges, as well as Indigenous cultural and intellectual property rights related to traditional foods, medicines, and health and wellness practices. Indigenous Peoples had data governance ecosystems and processes relevant to Indigenous knowledges before colonization.^21^ However, these systems are now being examined within a modern context. Indigenous data sovereignty and Indigenous data governance theory, practice and policy implementation are now ongoing in a few regions of the world, with Indigenous Peoples knowing best how to utilize data for the benefit of individual and community health and well-being (Box 1). Indigenous data sovereignty and governance, when enabled and supported, can contribute to the continued strengthening of Indigenous Peoples and their communities, and support them in making decisions and shaping policy based on their own needs, priorities and goals. Indigenous data sovereignty and governance can address and prevent the power imbalances and inequities that continue to be perpetuated by relevant policy-makers and the people who most often control data ecosystems and infrastructure relevant to Indigenous traditional medicine systems (for example, researchers, health systems).

Box 1 **Indigenous cultural and intellectual property processes in Australia**

The Australian continent has more than 6500 Indigenous edible plant species, 14 of which have been certified for commercial consumption.^22^ Currently, however, much of the governmental policy and legislation in Australia is framed according to settler-colonial terms and upholds neoliberal interests.^23^ Over recent years, Indigenous Peoples in Australia have been seeking avenues to explore the commercial and economic potential of place-based Indigenous knowledges, and Indigenous cultural and intellectual property rights related to traditional foods and medicines.^24^ Some refer to this endeavour as bush tucker sovereignty;^25^ however, the commercial potential of up to 60 000 years of Indigenous knowledges related to traditional foods and medicine is yet to be realized. Given this situation, the ongoing lack of rights and protections for Indigenous data sovereignty, Indigenous data governance, and Indigenous cultural and intellectual property rights, means Indigenous knowledges, including Indigenous traditional medicine and food practices, continues to be at risk of exploitation and appropriation.^26^ As a response to calls from Indigenous communities, the Australian government has established the Aboriginal and Torres Strait Islander Expert Working Group on Indigenous Cultural and Intellectual Property, which is chaired by Dr Terri Janke, a Wuthathi, Yadhaigana and Meriam woman. This working group is charged with ensuring that Indigenous knowledges, expertise and lived experience underpin the development of new legislation aimed at protecting Indigenous cultural and intellectual property rights. Indigenous Peoples and communities must lead these processes as they offer a wealth of knowledge that could be used to improve the understanding of the health and wellness benefits related to the use of traditional foods and bush medicine.

Several other pivotal Indigenous collectives are leading aligned work in Australia. The Lowitja Institute’s Indigenous Data Sovereignty Readiness Assessment and Evaluation Toolkit is designed to “improve the capabilities and processes of individuals through a whole-of-organization approach to embedding Indigenous data sovereignty in practice.”^27^^,^^28^ Additionally, *Maiam nayri Wingara* is a multidisciplinary collective of 12 leading Aboriginal and Torres Strait Islander scholars who are working with government, nongovernment and community organizations to embed Indigenous Data Governance processes in practice.^29^ Lastly, the Indigenous Data Network is a collective led by experts and stakeholders with a set of partnerships between the University of Melbourne, the Australian National University and the Australian Institute for Aboriginal and Torres Strait Islander Studies. The Indigenous Data Network is working to utilize developments in data sciences to maximize and optimize the ethical collection of, access to and use of data for community empowerment.^30^ All of these collectives are led by Indigenous Peoples for their peoples and communities. They all recognize that justice can only be achieved by working in equitable partnership with Indigenous Peoples and communities. This cumulative work can help to inform Indigenous data sovereignty, Indigenous data governance and Indigenous cultural and intellectual property rights and policies relevant to Indigenous traditional medicine in other contexts.

The challenges and issues surrounding Indigenous traditional medicine systems must be acknowledged to be based on the reality of stark colonial-driven health inequities and poverty among Indigenous communities globally. Indigenous Peoples make up about 6% of the world’s population, are three times as likely to live in extreme poverty and have the lowest life expectancy globally, as much as 20 years shorter than non-Indigenous Peoples.^31^^,^^32^ The increasing recognition of the importance and contribution of Indigenous traditional medicine systems to health, well-being and people-centred health care^33^ is integral to Indigenous Peoples’ health and well-being. Indigenous Peoples’ access to their traditional medicine systems will continue to depend on their access to self-determination, land and cultural rights, as well as Indigenous data sovereignty. Some of these issues are outlined in the following case studies relevant to Indigenous Peoples and their traditional medicine systems.

### Forced land eviction, East Africa

Forced land evictions have been both a historical and modern reality for many Indigenous Peoples in East Africa. Fortress conservation efforts that promote the removal of Indigenous Peoples from their lands continue today under various agendas,^34^ including carbon credit schemes.^7^ These human rights violations have ripple effects on all aspects of Indigenous Peoples’ lived experience, including the availability of and access to their traditional medicines. For example, the use of Indigenous traditional medicine among the Ogiek Indigenous community of Kenya has been integral to the health and well-being of community members for millennia. Traditional medicines such as medicinal plants are collected by individuals who have been gifted with healing hands and are therefore the only people who are permitted to collect, process and administer traditional treatments to patients. More often than not, critical illnesses are supported and treated by Indigenous traditional specialists within the community who are well known and respected herbalists or traditional healers. Support for children, fertility challenges and the work of birth attendants or midwives are often undertaken by women traditional healers; indeed, women in the Ogiek are seen to have the upper hand in traditional and medicinal plants. Despite intergenerational challenges in passing on Indigenous traditional medicine knowledge, after the coronavirus disease 2019 pandemic, community interest in learning about plant medicine increased, particularly among the younger generations. This change has resulted in more formalized efforts to transmit community knowledges. However, this process has been hampered by ongoing forced land evictions which have resulted in Ogiek communities being disconnected from their traditional lands, which are necessary both for access to traditional medicine and for intergenerational knowledge transfer.

For the Batwa Indigenous community in Uganda, their forced land eviction in the 1990s due to misguided conservation efforts has resulted in decades of land disconnection and therefore decades of forced disconnection from their traditional medicines and foods.^35^ Batwa Peoples have even been arrested for picking their own traditional medicines due to the militarization of the conservation agenda, with governments and conservation groups failing to uphold basic Human Rights and the Rights of Indigenous Peoples.^35^^,^^36^ Given this situation, for the Batwa to benefit from the World Health Organization’s (WHO) All for Health, Health for All initiatives, health agendas need to be more formally linked to human rights, land rights and Indigenous rights. The current denial of Batwa cultural and land rights denies them of their traditional medicines and foods, leading to substantial health inequities. Research highlighted a Batwa community member’s perspective who stated that:

When we left the forest, we started suffering and struggling with diseases because we could not get the local medicines we used to get from our forest of birth…we miss our strong medicines that we used to take while in the forest, because every time we would take the medicines we would be strong and happy.^35^

### Climate change, Peru

Climate change and its related impacts disproportionally affect Indigenous Peoples around the world.^37^^,^^38^ Indigenous Peoples’ close connection to land, water and ecosystems puts them at particular risk of a changing climate. These risks include the ability to pass on Indigenous knowledges, such as traditional medicine knowledge, which are in many cases dependent on the health of the ecosystem.^32^ For example, the climate change crisis is a serious environmental challenge for the medicine and health systems of Indigenous Peoples in the Peruvian Amazon. Changes in seasonality and more frequent extreme weather events^39^ are harming the environment and threatening biodiversity, which directly affects Indigenous medicines.^40^^,^^41^ Indigenous medicine systems depend on local natural resources, such as plants and animals collected from small farms or nearby mountains. Indigenous Peoples in the region also see forests as sources of food, medicine and other important resources or materials for their daily lives. Indigenous research in Peru highlighted a participant’s voice who stated that:

When we eat [our Shawi foods], we are happy. However, these days, we feel sad and think that fish will disappear. We think that in the future, we will have everything from our gardens, but not fish.^39^

Indigenous Peoples in Peru have been resilient to social and environmental change for centuries, as reflected in the more than 40 different Indigenous ethnic groups speaking their own languages. Climate change is a new threat, however, which is not being sufficiently addressed in the assumed dominant narratives of what constitutes a health-care system. Despite an intercultural health policy adopted in Peru, understanding among the governmental sector is still lacking on how to respectfully work with Indigenous Peoples and their ecological knowledge to adapt to climate change. Addressing this existential climate change challenge therefore requires not only policies to reduce health inequities in the region, but also a general policy recognition of Indigenous Peoples and their knowledges, including traditional medicine knowledge, to ensure the long-term health and well-being of the people and all facets of their ecological community. Overall, from “both an applied and philosophical sense, a health system not working within the planet’s own health boundaries…could be said to be antithetical to its entire purpose. Indigenous Peoples have known the concept of ecosystem and earth system boundaries within traditional health systems for millennia.”^42^ These views need to be respected and acted upon within wider health systems.

## The path forward

The inherent rights of Indigenous Peoples outlined in the United Nations Declaration on the Rights of Indigenous Peoples are clear. Yet, there are inconsistencies in how these rights are implemented around the world due to a lack of policy recognition of Indigenous Peoples as well as violations of their land rights.^43^ Many articles of the Declaration are relevant to the stewardship and practice of Indigenous traditional medicine systems (for example, Articles 3, 5, 11, 12, 13, 20, 21, 24, 25 and 26); however, Article 24 highlights specifically that:

Indigenous Peoples have the right to their traditional medicines and to maintain their health practices, including the conservation of their vital medicinal plants, animals and minerals. Indigenous individuals also have the right to access, without any discrimination, to all social and health services.^43^

With continued violations of Indigenous rights occurring around the world, the path towards universally achieving the rights in Article 24 is complicated. Yet, there is potential for regional, national, and international leadership and collaboration to better reinforce the importance of Indigenous traditional medicine systems for realizing universal health coverage and Health for All.^44^ Some examples are emerging within national contexts that could be considered for the development of wise practices, including in the Plurinational State of Bolivia and Nicaragua.^45^^,^^46^ Regardless, as global traditional medicine agendas become more entrenched, and with a particular focus on data-driven and evidenced-based processes, there is a continued risk of moving away from the key tenets that drive Indigenous traditional medicine systems. Given this risk, several key elements need to be understood to ensure that the conceptualization of health and well-being of Indigenous Peoples and their Indigenous traditional medicine systems are maintained for individual, community and planetary health. We briefly review these elements below.

### Important elements

Indigenous traditional medicine systems are non-homogeneous, with substantial diversity existing across the globe; however, most are underpinned by strong connections to Land and Country. Indigenous traditional medicine systems are most often spirituality driven, with a deep appreciation for the holistic and dynamic balance between humans and the more-than-human world, including Mother Earth as a whole, as well as the cosmos (for example, Father Sun, Grandmother Moon). Indigenous traditional medicine systems are evidence-informed based on millennia of empirical practice; are contextual, holistic, symbolic, nonlinear and relational; are not limited by time; are reliant on ecological health and balance; and are based on the collective observation of its people which explains healing phenomena through real and metaphoric narratives.^47^ Thus, Indigenous traditional medicine systems cannot be expected to be graded, categorized, standardized or boxed into Euro-Western scientific models and evidence hierarchies (that is, not subjected to conventional standards of practice). These key understandings about Indigenous traditional medicine systems underpin how health and well-being are conceptualized and practised within many Indigenous communities.

### Definition of health

Overall, conventional biomedical health systems have generally been devoid of planetary health considerations, with elements such as biodiversity rarely seen as being a relevant consideration for health-system functioning.^48^ This omission creates a problematic cycle for Indigenous Peoples and their traditional medicine systems, as a lack of land rights and therefore biodiversity protections further minimizes Indigenous Peoples’ ability to advocate for and protect their medicines, which has an impact on intergenerational health.^9^ The current definition of health itself, outlined by WHO (Box 2), contrasts with Indigenous views of health and well-being, which are tied to and interconnected with the health of Mother Earth. We developed an adaptation of the WHO definition of health informed by our own Indigenous perspectives (Box 2). Two authors drafted adapted versions which all of us then reviewed and edited until a consensus was reached within the group. We wanted to explicitly acknowledge that human survival is interconnected with Land and Country and is therefore dependent on a healthy environment. This understanding is crucial for the health of both Indigenous Peoples and non-Indigenous Peoples.

Box 2 **WHO definition of health and an adapted definition of health from our Indigenous community-based perspective**

**WHO definition**

Health is a state of complete physical, mental and social well-being and not merely the absence of disease or infirmity.^49^

**Our adapted definition**

Health is a state of dynamic balance within and between our physical, mental, social and spiritual well-being that is completely dependent on planetary health.

WHO: World Health Organization.

### Interconnectedness

Our adapted definition of health is informed by our own reflections and highlights a deeper understanding of the interconnectedness of health, culture and the environment, which fosters a more inclusive approach to health promotion and policy development. There has never been a greater urgency to preserve and protect Indigenous knowledges, than now, given the compounding crises of climate change, violations of Indigenous land rights and the scientific hegemony that continues to underpin health-care delivery while devaluing Indigenous traditional medicine practices and providers in many contexts.^5^ A more balanced approach is needed going forward that prioritizes Indigenous Peoples and their traditional practices, given their importance in bringing back ecological harmony to people, communities and the planet. These practices include Indigenous cultural and language revitalization, with support for ongoing intergenerational knowledge transfer. Indigenous land rights, access to traditional foods and the safeguarding of Sacred sites are integral to both Indigenous health outcomes and also to the health of all, as Indigenous Peoples are a key determinant of planetary health.^47^ When Indigenous Peoples have their health and well-being, they will be able to continue sustainably caring for more than a third of the world’s old-growth forests and the most biodiverse regions on the planet, and this ecological stewardship is beneficial to all.^47^

### Conclusion

In summary, current health-care systems are based on structural contexts and processes formed by colonization and they continue to maintain a separation between individuals, communities and the planet.^1^ Given this situation, Indigenous Peoples and their knowledge systems can provide “important viewpoints for health systems by bringing the health of the planet back into focus, emphasizing the importance of working together, context, and maintaining and learning from Indigenous ways of knowing that are innately connected to the Land.”^50^ Indigenous Peoples’ conceptualization of health has the potential to create a path forward towards harmony for people and health systems; however, this process will be dependent on ensuring an environment of reconciliation and good relationships, as well as an “acknowledgment of the harm that has been inflicted on Indigenous communities in the past and present context (that is, truth before reconciliation).”^50^ With this paper, we as Indigenous Peoples from around the globe highlight the importance of listening to and amplifying Indigenous voices within health, practice and policy spaces. At the same time, the need for free, prior and informed consent in any initiative, policy or practice that involves Indigenous Peoples and their traditional medicine systems (including traditional medicine providers) must be respected; in other words, “nothing about us, without us.”^11^
